# Supplementary material for: Differences in Acute Expression of Matrix Metalloproteinases-9, 3, and 2 Related to the Duration of Brain Ischemia and Tissue Plasminogen Activator Treatment in Experimental Stroke
Source: Int J Mol Sci. 2024 Aug 30;25(17):9442. doi: 10.3390/ijms25179442 (PMC11394866; doi:10.3390/ijms25179442)
Supplement: Supplementary file 1 [file ijms-25-09442-s001.zip › ijms-3152569-supplementary.pdf]

International Journal of
Molecular Sciences

MDPI

Supplementary Table S1. Percent of total variation and significance of interactions among the variables (ischemic time, hemisphere, and treatment) in the three-way ANOVA for MMP expression.

	**Interactions**							
	**Ischemic time X Hemisphere**		**Ischemic time X Treatment**		**Hemisphere X Treatment**		**AxBxC**	
	P value	%TV	P value	%TV	P value	%TV	P value	%TV
**MMP-9**	0.0191	2.5	<0.0001	10.6	0.0011	3.5	0.0165	2.6
**MMP-2**	0.0009	12.7	0.0045	9.4	0.0073	6.1	0.1388	3.2
**MMP-3**	0.501	1.0	0.1454	3.0	0.7505	0.1	0.3311	1.7
